# Development and effects of a scenario-based labor nursing simulation education program using an artificial intelligence tutor: a quasi-experimental study

**DOI:** 10.4069/whn.2025.06.18

**Published:** 2025-06-30

**Authors:** Seo-A Park, Hye Young Kim

**Affiliations:** 1 College of Nursing, Kyungwoon University, Gumi, Korea; 2 College of Nursing, Keimyung University, Daegu, Korea

**Keywords:** Artificial intelligence, Education, Nursing, Obstetric nursing, Simulation training

## Abstract

**Purpose:**

This study was conducted to determine the effects of a scenario-based labor nursing simulation education program using an artificial intelligence (AI) tutor on knowledge, critical thinking disposition, clinical performance abilities, and digital literacy.

**Methods:**

This quasi-experimental study employed a non-equivalent control group pre- and post-test design. Seventy-two fourth-year nursing students (38 experimental, 34 control) were recruited from a university in Gumi, Korea. The experimental group participated in a scenario-based simulation education program supported by an AI tutor, while the control group engaged in conventional high-fidelity simulation training. Both groups attended sessions once per week for 2 hours over five sessions. Data were collected from August to December 2023 and analyzed using descriptive statistics, homogeneity testing, and the independent t-test.

**Results:**

Simulation using an AI tutor resulted in significantly higher scores for the experimental group compared to the control group in nursing knowledge (t=7.03, *p*<.001), clinical performance (t=7.80, *p*=.020), and digital literacy (t=5.02, *p*<.001).

**Conclusion:**

The scenario-based labor nursing simulation program utilizing an AI tutor was effective in improving nursing students’ delivery of knowledge, clinical performance, and digital literacy. Future research should focus on developing diverse and advanced simulation programs tailored to various areas of women’s health nursing, to further enhance critical thinking and clinical judgment capabilities.

## Introduction

In nursing education, the importance of simulation-based learning has become increasingly emphasized. Simulation has been widely recognized as an effective instructional strategy for enhancing learners’ clinical performance, critical judgment, and self-confidence [1,2]. It allows students to experience complex clinical situations in advance and improve their skills through experiential learning [3,4]. Labor and delivery nursing, in particular, involves high-risk situations requiring refined judgment and advanced technical skills, making exposure to such environments essential during training [5,6]. However, actual clinical practice often limits these opportunities due to privacy concerns, safety regulations, and constraints of time or space [7,8]. High-fidelity simulation (HFS) has been utilized to address these limitations, but its dependence on instructor guidance and resource demands can hinder consistent implementation [2].

With the rapid advancement of digital technologies, the adoption of artificial intelligence (AI) in education has expanded significantly. AI-based educational methods are especially effective for maximizing learning outcomes by enabling personalized and adaptive learning tailored to individual learners’ levels and characteristics [9-11]. Chatbots, as conversational AI tools, create a learner-centered and interactive environment by engaging students in real-time question-and-answer exchanges [9-12]. Unlike traditional AI systems that provide static, unidirectional information, conversational chatbots simulate dynamic dialogue and adaptive feedback based on user input, enabling personalized and responsive learning. This interactive approach encourages learners to seek clarification, articulate clinical reasoning, and reinforce understanding through repeated engagement with scenario-driven content.

In line with these trends, the field of nursing education has begun to explore AI-integrated simulation as an innovative alternative to traditional instructional approaches. Simulation education utilizing an AI tutor provides a means to overcome the limitations of conventional teaching formats, where instructors must guide multiple learners simultaneously with limited resources [12-14]. AI tutors allow nursing students to access essential information in real time and engage in individualized learning processes aligned with their unique academic levels and needs [12,13]. These features contribute to greater learner autonomy and improved educational efficiency. Generation Z learners, who are naturally attuned to digital environments, further support the adoption of AI-enhanced strategies [15], which can improve knowledge, critical thinking, clinical competency, and digital literacy [9-11,16]. This digital affinity strengthens the case for incorporating AI tutor-assisted simulation into nursing curricula [12,15,17]. In the context of nursing simulation, scenario-based conversational chatbots can present structured clinical cases, guide learners through stepwise decision-making, and provide immediate feedback aligned with educational objectives. These features reduce instructors’ workload while fostering active learning, knowledge retention, and clinical reasoning. By delivering consistent, self-paced content, conversational chatbots improve the flexibility and accessibility of simulation education without compromising quality [18-20]. To meet the needs of today’s rapidly evolving educational landscape, nursing curricula must support students in linking theory to practice and building clinical adaptability.

Accordingly, the present study aims to develop and evaluate a scenario-based AI tutor-assisted labor nursing simulation education program. By implementing this program through a chatbot system based on instructor-designed scenarios, the study seeks to overcome the limitations of traditional didactic instruction and existing simulation environments, while promoting learner-centered education and improving clinical performance competency.

The aim of this study was to assess the impact of a scenario-based labor nursing simulation program, supported by an AI tutor, on fourth-year nursing students’ knowledge, critical thinking, clinical performance, and digital literacy.

The research hypotheses were as follows:

• Hypothesis 1: There would be differences in nursing knowledge between the experimental group, which participated in the scenario-based labor nursing simulation education program using an AI tutor, and the control group.

• Hypothesis 2: There would be differences in critical thinking disposition between the experimental group and the control group.

• Hypothesis 3: There would be differences in clinical performance between the experimental group and the control group.

• Hypothesis 4: There will be differences in digital literacy between the experimental group and the control group.

## Methods

**Ethics statement:** This study was approved by the Institutional Review Board of Kyungwoon University (No. KW-2023-A-4). Informed consent was obtained from the participants.

### Research design

A non-equivalent pre-test/post-test design was employed to analyze the effects of a scenario-based labor nursing simulation education program using an AI tutor on labor nursing knowledge, critical thinking disposition, clinical performance, and digital literacy among nursing students. This study adhered to the TREND (Transparent Reporting of Evaluations with Nonrandomized Designs) statement guidelines for quasi-experimental studies [21].

### Sample and sampling

Fourth-year nursing students enrolled at Kyungwoon University in Gumi, Korea, were recruited by posting advertisements created by an independent research assistant who was not involved in student grading. The researcher directly explained the purpose of the study to prospective participants, clearly stating that participation in the study was not part of the regular academic curriculum. Students who agreed to participate were provided with a questionnaire, and it was emphasized that participation was voluntary and that they could withdraw at any time without penalty. Participants were informed that their responses would remain anonymous and would be used solely for research purposes. The required sample size was determined based on a previous study [22], which examined the effects of HFS education on clinical performance among nursing students and reported an effect size of 0.88. Using G*Power ver. 3.1.0 [23], the required sample size for comparing means between two groups was calculated using a significance level (α) of .05, statistical power (1–β) of .80, and an effect size (d) of .88. Based on these parameters, a total of 44 participants (22 per group) was initially required; considering a 30% dropout rate, the target sample size was adjusted to 32 per group. However, due to high interest and voluntary participation, a total of 72 nursing students were ultimately recruited. Students indicating voluntary agreement to participate were included through convenience sampling. Group assignment was performed using the Microsoft Excel (Microsoft Corp., Redmond, WA, USA) program, resulting in 38 students in the experimental group and 34 in the control group.

### Instrument

#### Knowledge of labor nursing

Knowledge of labor nursing was assessed using a questionnaire developed by the researcher, based on the learning objectives of maternal nursing and relevant literature [22]. The 15 items cover core knowledge related to labor nursing, and each item is scored as 1 point for a correct answer and 0 points for an incorrect or “don’t know” response. Total scores range from 0 to 15, with higher scores indicating greater knowledge. In this study, the reliability of the instrument, as measured by Cronbach’s α, was .83.

#### Critical thinking disposition

Critical thinking disposition was measured using an assessment tool developed by Yoon [24] to facilitate problem-solving and decision-making among nursing students, with the author’s permission. The tool consists of 27 items across seven subscales: intellectual curiosity (five items), prudence (four items), confidence (four items), systematicity (three items), intellectual fairness (four items), healthy skepticism (four items), and objectivity (three items). Each item is rated on a 5-point Likert scale, from 1 (“strongly disagree”) to 5 (“strongly agree”), yielding a total possible score from 27 to 135. Higher scores indicate higher critical thinking disposition. In Yoon’s original study [24], Cronbach’s α was .84; in the present study, Cronbach’s α was .81.

#### Clinical performance

Clinical performance was measured using a tool originally developed by Lee et al. [25] for nursing students, later revised and supplemented by Choi [26], and used here with permission. This 45-item tool measures five domains of clinical performance: professional development (nine items), nursing skills (11 items), nursing education/collaboration (eight items), interpersonal relationships/communication (six items), and nursing process (11 items). Each item is rated on a 5-point Likert scale, ranging from 1 (“very poor”) to 5 (“very good”), with higher scores (possible score range, 45–225) indicating higher levels of clinical performance. The reliability of the original tool was demonstrated by Cronbach’s α values of .96 in its original development [25], .92 for its supplemented version [26], and .88 in the present study.

#### Digital literacy

The digital literacy subscale from the tool developed by Lee [27] to assess digital literacy among university students was used, with the author’s permission. The full instrument contains 70 items assessing digital ethics and literacy across four subscales: digital citizenship ethics (19 items), digital etiquette (17 items), digital literacy (18 items), and digital norms (16 items). Only the 18 digital literacy items were used in this study. Each item was rated on a 5-point Likert scale from 1 (“strongly disagree”) to 5 (“strongly agree”), for a total score range of 18–90. Higher scores indicate higher digital literacy. Reliability for the digital literacy subscale was shown by Cronbach’s α values of .92 in its development [27] and .93. in the present study.

#### General characteristics

General characteristics included participants’ age, gender, degree of self-expression, satisfaction with their major and clinical practice, interpersonal relationship level, methods of obtaining information, perceived need for AI education, and overall perceptions of AI education.

### Research procedures

#### Development of the scenario-based labor nursing simulation education program using an AI tutor

This study was conducted in four phases—analysis, design, implementation, and evaluation—following the System Development Life Cycle framework to create a scenario-based, AI tutor-assisted labor nursing simulation education program [28].

In the analysis phase, the educational program’s goals were established, educational needs were assessed, and an expert group was organized to guide the development of the scenario-based AI tutor-assisted labor nursing simulation program.

The educational content was structured using the researcher’s previously developed HFS modules, covering key topics in obstetric nursing. These included assessment of uterine contractions and the labor process, measurement of fundal height, application of the non-stress test, interpretation of fetal heart rate monitoring, Leopold’s maneuvers, pain relief techniques, breathing techniques, and psychological support for the mother and family [22].

During the design phase, learning objectives and content structure were developed based on the analysis phase findings. The dialogue flow and instructional messages that the AI tutor chatbot would deliver for the scenario-based simulation were designed. The screen layout, visual design, and scenes for the scenario-based AI tutor chatbot simulation were planned. To validate the developed AI-based educational program, a panel of three nursing professors and one AI program expert evaluated its educational validity. The AI chatbot prototype was developed in consultation with a chatbot development expert, using the Danbee platform (https://danbee.ai/), which offers advanced natural language processing and Korean language specialization [29]. To assess nursing students’ educational needs, learners completed pre-learning inquiries and educational needs questionnaires, structured in Microsoft Excel and integrated into the AI tool. The learning interface included video links, clickable buttons, and pop-up functionalities. The AI platform distinguished among simulation orientation, pre-learning, and main learning phases, enabling real-time interaction with the AI tutor by means of questions and answers.

In the implementation phase, a prototype chatbot was developed with AI prompts specifying learning objectives, phases, and constraints to ensure accurate educational support. The chatbot’s performance was optimized through iterative testing and refinement. A pilot operation was conducted with three nursing professors, who received a comprehensive guide, including an overview, operational procedures, AI prompts, and demonstration examples, to support implementation.

**Figure 1.**
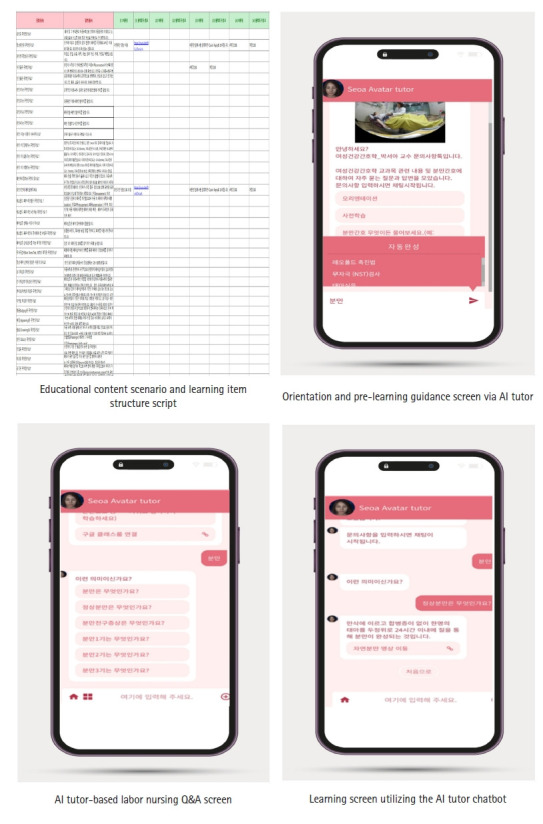
Labor nursing simulation education program using an artificial intelligence (AI) tutor program.

In the evaluation phase, the chatbot’s usability was assessed by surveys and interviews with nursing students, and improvements were incorporated. The final platform was structured into three stages: simulation orientation, pre-learning, and main learning, with real-time AI tutor support at each stage. The iterative process refined the system, resulting in the final AI chatbot (Figure 1).

#### Implementation of the labor nursing simulation program using an AI tutor

The simulation education implemented in this study utilized the SimMom Laerdal HFS developed by Laerdal Medical AS (Stavanger, Norway). This study was conducted from August 28 to December 31, 2023.

The program was structured to accommodate the simulation laboratory environment and the number of participants. Simulation-based sessions were conducted individually and in groups, once per week for 2 hours across five sessions, following the HFS education framework. In the first week, a 2-hour theoretical lecture was provided, covering essential maternal nursing topics, including uterine contractions, labor components, mechanisms of labor, pain and discomfort assessment, fetal electronic monitoring, and simulation practice procedures. During the second and third weeks, 72 students (experimental and control groups) participated in group-based practical training. Students utilized the HFS or participated in role-playing exercises as nurses and patients to improve their understanding and hands-on practice of labor scenarios. In the fourth week, group-based practice sessions using the SimMom simulator were held, with each simulation lasting approximately 15 minutes. The researcher provided preliminary explanations of the simulation room, simulator functions, and materials before facilitating the scenarios. Both groups used the HFS, with additional patient information provided by faculty during simulations to enrich learning. The fifth week was a debriefing session where all students gathered to reflect on their experiences, discuss strengths and weaknesses, summarize scenarios, and analyze patient care approaches. Feedback and advice supported learning integration (Table 1).

The experimental group received HFS integrated with the scenario-based AI tutor. Their training included presentation of objectives, pre-learning with the AI tutor, simulation practice, and debriefing. Pre-learning activities were accessed via QR code (https://danbee.ai/), allowing students to ask questions and receive answers through the chatbot. The chatbot matched user questions to responses from a pre-accumulated database of learning outcomes.

To prevent diffusion of intervention effects, it was explained that although the lecture content during the first session was the same for both the experimental and control groups, the instructional methods differed. To minimize the risk of selection bias, participants were randomly assigned to groups using IBM SPSS for Windows, ver. 25.0 (IBM Corp., Armonk, NY, USA). To avoid contamination between participants before and after the intervention, students were instructed to remain in separate areas—specifically, a waiting room prior to the intervention, the simulation center during the intervention, and a post-intervention waiting room.

The control group participated in conventional HFS education without the AI tutor intervention, for the same period of 5 weeks. During the evaluation phase, the effectiveness of the scenario-based AI tutor-assisted labor nursing simulation program was assessed. Participants completed a pre-test measuring nursing knowledge, critical thinking disposition, clinical performance competency, and digital literacy prior to program implementation, and a post-test was administered immediately after program completion (Table 1).

During questionnaire completion, the researcher vacated the room to avoid influencing participant responses. Additionally, after the entire simulation education program was completed, the control group was also provided with the same educational program as the experimental group. Upon completion of the survey, each participant received a small gift—a ballpoint pen valued at approximately 7.40 US dollars—as a token of appreciation.

### Data analysis

The data collected in this study were analyzed using IBM SPSS for Windows, ver. 25.0, as follows:

1) To test the study hypotheses, comparisons between the experimental and control groups were conducted using two-tailed tests, with statistical significance set at *p*<.05. Descriptive statistics, including frequency, percentage, mean, and standard deviation, were used to summarize participants’ general characteristics.

2) The independent t-test was used to assess homogeneity in general characteristics between the two groups.

3) The independent t-test was used to compare the two groups regarding knowledge of labor nursing, critical thinking, clinical performance, and digital literacy.

## Results

### Participant characteristics and homogeneity testing

The results of the homogeneity test for general characteristics between the experimental and control groups indicated no statistically significant differences, confirming that the groups were homogeneous (Table 2).

The mean age was 23.36±1.56 years in the experimental group and 23.55±1.59 years in the control group. In the experimental group, 10.5% of participants were male and 89.5% were female, while in the control group, 17.6% were male and 82.4% were female.

For self-expression, mean scores were 2.37±0.82 in the experimental group and 2.18±0.97 in the control group. Satisfaction with the nursing department was 2.21±0.87 in the experimental group and 2.02±0.80 in the control group, while satisfaction with clinical practice was 2.53±0.95 and 2.58±0.96, respectively. Human relationships scored 1.97±0.68 in the experimental group and 1.82±0.80 in the control group. Regarding methods of acquiring information, 47.4% of the experimental group reported using textbooks, 2.6% used the internet, and 50.0% consulted professors. In the control group, 41.2% used textbooks, 5.9% used the internet, and 52.9% obtained information from professors. Regarding the need for AI education, 60.5% of the experimental group and 70.6% of the control group answered “yes.” In terms of awareness of AI education, 25.0% of the experimental group responded “well,” 69.4% “moderately,” and 5.6% “not at all.” In the control group, the responses were 19.4% “well,” 63.9% “moderately,” and 16.7% “not at all.”

Regarding baseline measures, the experimental group had a mean knowledge of labor nursing score of 13.18±2.42 out of a possible 15 points, while the control group scored 12.20±2.44; this difference was not statistically significant (t=1.70, *p*=.725). For critical thinking disposition (possible range, 27–135), the experimental group scored 60.18±11.20, and the control group 58.44±11.17, indicating a moderate level in both groups with no significant difference (t=0.66, *p*=.946). Clinical performance competency (possible range, 45–225) was slightly higher in the control group (169.20±21.13) compared to the experimental group (161.71±23.39); however, this difference was not statistically significant (t=–1.42, *p*=.406). For digital literacy (possible range: 18–90), the experimental group reported a mean of 71.97±12.13, while the control group scored 68.23±15.05, with no statistically significant difference (t=1.16, *p*=.344).

### Hypothesis testing

The results of hypothesis testing for the effects of the scenario-based AI tutor-assisted labor nursing simulation program on labor nursing knowledge, critical thinking disposition, clinical performance competency, and digital literacy are presented as follows (Table 3).

#### Hypothesis 1

The post-intervention mean score for labor nursing knowledge was 14.78±0.62 in the experimental group and 12.20±2.32 in the control group. The difference was statistically significant (t=7.03, *p*<.001), supporting Hypothesis 1, which posited that labor nursing knowledge would be higher in the experimental group.

#### Hypothesis 2

The post-intervention mean score for critical thinking disposition was 62.31±9.43 in the experimental group and 58.73±7.42 in the control group. The difference was not statistically significant (t=1.77, *p*=.098); thus, Hypothesis 2, which posited higher critical thinking disposition in the experimental group, was not supported.

#### Hypothesis 3

The post-intervention mean score for clinical performance competency was 203.62±13.56 in the experimental group and 171.11±20.74 in the control group. The difference was statistically significant (t=7.80, *p*=.020), supporting Hypothesis 3, which posited that clinical performance competency would be higher in the experimental group.

#### Hypothesis 4

The post-intervention mean score for digital literacy was 82.00±6.80 in the experimental group and 71.23±13.53 in the control group. This difference was statistically significant (t=4.18, *p*<.001), supporting Hypothesis 4, which posited that digital literacy would be higher in the experimental group.

## Discussion

This study evaluated the effects of a 5-week scenario-based AI tutor-assisted labor nursing simulation education program using a chatbot on labor nursing knowledge, critical thinking disposition, clinical performance competency, and digital literacy among nursing students.

The greater improvement in labor nursing knowledge observed in the experimental group supports the effectiveness of scenario-based AI tutor-assisted simulation education. This finding aligns with previous research [22], which has shown that HFS contributes to enhanced nursing knowledge by enabling students to engage in realistic, repetitive clinical scenarios. Simulation-based education has also been widely reported to improve knowledge acquisition, procedural skills, and performance, particularly in high-risk areas of obstetrical nursing [30,31]. In this study, the integration of an AI tutor enabled individualized feedback and repeated engagement with learning materials [20], which likely deepened students’ understanding. This supports the findings of Buchanan et al. [13], who emphasized that AI-driven personalized education promotes the transfer of knowledge in nursing education. These outcomes suggest that future nursing education programs may benefit from incorporating chatbot-based AI tutors, particularly in areas requiring high levels of clinical reasoning and adaptability.

Although the experimental group showed a higher mean score for critical thinking disposition compared to the control group, the difference was not statistically significant. This finding differs from previous studies, such as Park and Kim [22], which reported improvements in critical thinking following labor nursing simulation-based education, and Song [31], which also demonstrated enhanced cognitive outcomes through general integrative simulation practice. However, it is consistent with the findings of Kim and Ha [30], who reported that although critical thinking disposition scores increased in the experimental group after simulation-based education on postpartum hemorrhage, the difference was not statistically significant.

Critical thinking requires sufficient knowledge to be applied in problem-solving situations, as well as continuous exposure to diverse scenarios through ongoing learning [22,31]. In this study, the relatively short intervention period and the use of a single educational module may have been insufficient to produce meaningful improvement in critical thinking disposition. Given that critical thinking development requires persistent and repeated problem-solving experiences [3], this finding suggests the need for a long-term educational approach. Additionally, there are growing concerns that the rapid expansion of AI-based education may intensify learners’ tendencies to accept information uncritically. While learning through an AI tutor offers convenience and efficiency, it poses the risk that learners may passively accept information presented by the system rather than actively recognizing and analyzing problems themselves [32].

AI-based learning environments may encourage passive information acceptance, which can undermine the development of critical thinking skills and weaken learners’ autonomous problem-solving abilities [33]. Moreover, overreliance on standardized answers provided by AI-based learning systems may limit adaptability in complex and creative problem-solving situations [34].

The experimental group that participated in the scenario-based AI tutor-assisted labor nursing simulation program demonstrated a statistically significant improvement in clinical performance competency compared to the control group. This result is consistent with previous studies verifying the effectiveness of simulation-based education programs for nursing students [22,30,31]. Simulation-based education is thought to enhance clinical performance competency by enabling students to acquire theoretical knowledge through pre-learning and by exposing them to clinical-like situations without actual clinical experience, thereby facilitating the development of problem-solving approaches to patient care. In the present study, the combination of acquiring foundational knowledge through the AI tutor and applying that knowledge during simulation practice appeared to deepen students’ understanding of clinical situations, likely contributing to the improvement in their clinical performance competency. Thus, the integration of AI tutor-based pre-learning with HFS practice may have strengthened the connection between theory and practice, resulting in more effective clinical skills development.

The experimental group also demonstrated a statistically significant improvement in digital literacy compared to the control group. Supporting this finding, Deveci et al. [11] reported that learning through chatbot applications enhances digital technology utilization skills and improves self-directed learning abilities. This suggests that AI-based learning environments can effectively foster digital literacy and learner autonomy in nursing students. These results suggest that the integration of AI-based educational tools into nursing education can contribute to strengthening learners’ self-directed learning competencies by providing immediate feedback, increasing opportunities for repeated learning, and facilitating autonomous exploration of learning content.

In particular, creating virtual patient scenarios that simulate clinical situations difficult to experience in actual practice can provide students with valuable opportunities to strengthen the critical thinking skills necessary for effective problem-solving. Simulation education has been reported to improve critical thinking disposition, clinical judgment, clinical performance competency, and problem-solving ability among nursing students. Moreover, simulation provides a realistic learning environment that has been shown to enhance learners’ confidence and satisfaction while reducing anxiety [22,31].

Accordingly, there is a growing need to develop a variety of simulation scenarios tailored to the specific characteristics of different areas within women’s health nursing. Such case-based simulations can help students prepare for potential challenges they may encounter by fostering their ability to prioritize and make clinical decisions in complex situations. Each scenario should be systematically designed to align with specific learning objectives, with the level of difficulty gradually adjusted. Additionally, providing individualized feedback through AI can support students in progressively building problem-solving skills, from novice to advanced levels, within a structured educational framework that integrates simulation and AI technologies [10,35,36]. During the simulation process, instructional strategies may also include the AI tutor prompting students with appropriate questions or hints to encourage reflective thinking and decision-making. By applying such systematic teaching-learning strategies, it is anticipated that students will not only acquire knowledge but also cultivate higher-order thinking skills, empowering them to think critically and make independent clinical judgments.

Efforts to ensure the sustainability of educational effects are essential. Previous studies have shown that repetitive and continuous simulation experiences contribute to ongoing improvements in knowledge acquisition, clinical performance competency, and critical thinking skills among students [36,37]. Thus, it is important to provide learners with opportunities for gradual and repeated practice, which facilitates long-term memory retention and enhances performance capabilities. Moreover, longitudinal follow-up studies are necessary to confirm the long-term effectiveness of newly developed educational programs. As AI technology continues to evolve, ongoing research and iterative refinement should accompany its integration into nursing education. Rigorous studies are needed to assess the effectiveness of simulation scenarios utilizing state-of-the-art AI algorithms, evaluate student acceptance, and address potential ethical considerations. At the same time, it is crucial to strengthen the competencies of nursing educators to ensure they can effectively implement and leverage these emerging technologies in educational environments [35].

This study has several limitations. First, because participants were recruited through convenience sampling from a single university, the generalizability of the findings may be limited. Second, the long-term sustainability of the effects of simulation-based education was not evaluated. Future research should therefore examine the long-term impact of simulation-based educational interventions over extended follow-up periods.

Despite these limitations, this study makes a meaningful contribution by developing and evaluating a labor nursing simulation education program utilizing an AI tutor for nursing students. By incorporating a scenario-based AI tutor, the program provided an innovative educational environment that allowed students to repeatedly access information and receive immediate feedback, unconstrained by time or place. This approach offers a more systematic and learner-centered experience compared to traditional simulation education. If such programs are implemented on a broader scale, it is expected that they will not only increase nursing students’ knowledge, problem-solving skills, and clinical performance but also establish a creative and effective educational model with positive impacts extending into clinical practice settings.

In conclusion, this study demonstrated that a scenario-based, AI tutor-assisted labor nursing simulation education program effectively improved labor nursing knowledge, clinical performance competency, and digital literacy among nursing students. Implementation of such programs can help maximize learner-centered educational outcomes, fostering greater engagement and deeper understanding. AI-based chatbots show strong potential for diverse applications in nursing education, including supporting simulation activities, enhancing knowledge acquisition, promoting skill development, and increasing learning retention and student engagement. It is therefore recommended that future women’s health nursing curricula strategically incorporate AI-based simulation programs to further enhance educational effectiveness and prepare students for digitally driven healthcare environments.

## Figures and Tables

**Table 1.** Research procedures

Session	Experimental group (n=38)	Control group (n=34)
1	• Pre-test	• Pre-test
	-General characteristics, knowledge of labor nursing, critical thinking disposition, clinical performance, digital literacy	-General characteristics, knowledge of labor nursing, critical thinking disposition, clinical performance, digital literacy
	• Orientation and prerequisite learning (2 hours)	• Orientation and prerequisite learning (2 hours)
	• AI tutor education	
2–3	• Scenario assessment (20 minutes)	• Scenario assessment (20 minutes)
	• Intervention training (40 minutes)	• Intervention training (40 minutes)
	• Team learning and role play (30 minutes)	• Team learning and role play (30 minutes)
	• Labor nursing simulation education program using an HFS (SimMom) (30 minutes)	• Labor nursing simulation education program using an HFS (SimMom) (30 minutes)
	• AI tutor education	
4	• Simulation running using HFS (SimMom)	• Simulation running using HFS (SimMom)
	• Recording worksheet (30 minutes)	• Recording worksheet (30 minutes)
	• Feedback and evaluation (60 minutes)	• Feedback and evaluation (60 minutes)
	• AI tutor education	
5	• Debriefing and evaluation	
	• Post-test	
	-Knowledge of labor nursing, critical thinking disposition, clinical performance, digital literacy	

Experimental group: a labor nursing simulation education program using an artificial intelligence (AI) tutor and a high-fidelity simulator (HFS). Control group: a labor nursing education program using an HFS.

**Table 2.** Homogeneity test of characteristics and variables between the experimental and control groups (N=72)

Characteristic/variable	Categories	n (%) or mean±SD		χ²/t	*p*
		Experimental group (n=38)	Control group (n=34)		
Age (year)		23.36±1.56	23.55±1.59	–0.50	.516
Gender	Male	4 (10.5)	6 (17.6)	0.85	.865
	Female	34 (89.5)	28 (82.4)		
Level of self-expression		2.37±0.82	2.18±0.97	0.91	.300
Satisfaction with nursing department		2.21±0.87	2.02±0.80	0.91	.173
Satisfaction with clinical practice		2.53±0.95	2.58±0.96	–0.15	.978
Human relationships		1.97±0.68	1.82±0.80	0.85	.144
Information acquisition methods	Textbooks	18 (47.4)	14 (41.2)	–0.39	.407
	Internet	1 (2.6)	2 (5.9)		
	Professors	19 (50.0)	18 (52.9)		
Need for AI in education	Yes	23 (60.5)	24 (70.6)	0.84	.105
	No	2 (5.3)	4 (11.8)		
Awareness of AI education	Well aware	9 (25.0)	7 (19.4)	–1.67	.514
	Moderate	25 (69.4)	23 (63.9)		
	Not aware at all	2 (5.6)	6 (16.7)		
Nursing knowledge		13.18±2.42	12.20±2.44	1.70	.725
Critical thinking disposition		60.18±11.20	58.44±11.17	0.66	.946
Clinical performance		161.71±23.39	169.20±21.13	–1.42	.406
Digital literacy		71.97±12.13	68.23±15.05	1.16	.344

AI, artificial intelligence.

**Table 3.** Comparisons of knowledge of labor nursing, critical thinking disposition, clinical performance, and digital literacy scores between experimental and control groups after the intervention (N=72)

Variable	Possible score range	Group	Mean±SD			t	*p*
			Pre-test	Post-test	Difference		
Knowledge of labor nursing	0–15	Experimental (n=38)	13.18±2.42	14.78±0.62	0.97±0.57	7.03	<.001
		Control (n=34)	12.20±2.44	12.20±2.32	2.76±0.41		
Critical thinking disposition	27–135	Experimental (n=38)	60.18±11.20	62.31±9.43	1.74±2.64	1.77	.098
		Control (n=34)	58.44±11.17	58.73±7.42	3.58±2.01		
Clinical performance	45–225	Experimental (n=38)	161.71±23.39	203.76±13.56	–7.49±5.27	7.80	.020
		Control (n=34)	169.20±21.13	171.11±20.74	32.64±4.08		
Digital literacy	18–90	Experimental (n=38)	71.97±12.13	82.00±6.80	3.73±3.2	4.18	<.001
		Control (n=34)	68.23±15.05	71.23±13.53	10.76±2.56		
